# Meta-analytic support vector machine for integrating multiple omics data

**DOI:** 10.1186/s13040-017-0126-8

**Published:** 2017-01-26

**Authors:** SungHwan Kim, Jae-Hwan Jhong, JungJun Lee, Ja-Yong Koo

**Affiliations:** 1 Department of Statistics, Korea University, Anam-dong, Seoul, 136-701 South Korea; 2 Department of Statistics, Keimyung University, Dalseoku, Daegu, 42601 South Korea

**Keywords:** Support vector machine, Meta-analysis, Data integration, TCGA

## Abstract

**Background:**

Of late, high-throughput microarray and sequencing data have been extensively used to monitor biomarkers and biological processes related to many diseases. Under this circumstance, the support vector machine (SVM) has been popularly used and been successful for gene selection in many applications. Despite surpassing benefits of the SVMs, single data analysis using small- and mid-size of data inevitably runs into the problem of low reproducibility and statistical power. To address this problem, we propose a meta-analytic support vector machine (Meta-SVM) that can accommodate multiple omics data, making it possible to detect consensus genes associated with diseases across studies.

**Results:**

Experimental studies show that the Meta-SVM is superior to the existing meta-analysis method in detecting true signal genes. In real data applications, diverse omics data of breast cancer (TCGA) and mRNA expression data of lung disease (idiopathic pulmonary fibrosis; IPF) were applied. As a result, we identified gene sets consistently associated with the diseases across studies. In particular, the ascertained gene set of TCGA omics data was found to be significantly enriched in the ABC transporters pathways well known as critical for the breast cancer mechanism.

**Conclusion:**

The Meta-SVM effectively achieves the purpose of meta-analysis as jointly leveraging multiple omics data, and facilitates identifying potential biomarkers and elucidating the disease process.

**Electronic supplementary material:**

The online version of this article (doi:10.1186/s13040-017-0126-8) contains supplementary material, which is available to authorized users.

## Introduction

Over the last decade, the technologies of microarray and massively parallel sequencing generate multiple omics sources from a large cohort at an unprecedented rate. Besides, since the experimental costs have dropped, a huge amount of data sets have been accumulated in public data repositories (e.g., Gene Expression Omnibus (GEO) and Sequence Read Archive (SRA)). And yet low reproducibility has been a chronic concern due to mid-and-small size of each individual experimental unit (e.g., 40–100) and low signal-to-noise ratios of genomic expression data [24, 26, 27]. In an effort to tackling these challenges, effective data integration methods have been widely spotlighted in biomedical research [2]. The traditional meta-analysis integrates significance levels or effect sizes of similar data sets (similar design or biological hypothesis), and has proven to be effective in discovering significant biomarkers [14, 37]. Multi-study data integration is also known as “horizontal meta-analysis” that combines multiple homogeneous omics data [38]. Moreover, many large consortia such as the Cancer Genome Atlas (TCGA) and Lung Genomics Research Consortium (LGRC) have generated different types of omics data (e.g., mRNA, methylation, CNV and so on) using samples from a single cohort. Datasets are aligned vertically by samples, and thus integration of such multi-omics data is called “vertical omics integrative analysis” [38]. Jointly leveraging multi-layers of omics data, vertical omics integration facilitates deciphering biological processes, capturing the interplay of multi-level genomic features, and elucidating how a priori knowledge of biological information (e.g., pathway database) functions within the framework of systems biology.

Generally high-throughput microarray and sequencing data have been extensively applied to monitor biomarkers and biological processes related to many diseases [4], to predict complex diseases (e.g., cancer diagnosis, [36]), prognosis [45], and therapeutic outcomes [23]. In particular, the recent classification and prediction tools have notably advanced the translational and clinical applications (e.g. MammaPrint [43]), Oncotype DX [30] and Breast Cancer Index BCI [49]. In this trend, the support vector machine (SVM) has been also popularly applied to many genomic applications and proved as one of the most powerful prediction methods [3, 15, 29] attributed to unmatched flexibility of non-linear decision boundary. Commonly gene selection (a.k.a. feature reduction) pertaining to outcomes diminishes the dimension of expression data, enabling to shorten the training time and to enhance interpretability. In addition, gene selection removes a large number of irrelevant genes that potentially undermine precise prediction, and notably the idea of feature selection using SVMs can extend to the setting of multi-omics data analysis ([18, 25]). As this concern related, many researchers have put tremendous efforts to circumvent low accuracy of the SVMs when analyzing high-dimensional genomic data. For instance, Brown et al. [5] introduced a functional gene classification including the usage of various similarity functions (e.g., kernels modeling prior knowledge of genes). Moreover, as SVM takes on the small subset of samples that differentiate between class labels with an exclusion of the remaining samples, it is believed to have the potential to handle large feature spaces and the ability to identify outliers. Guyon et al. [9] also proposed a gene selection method that utilizes the SVM based on Recursive Feature Elimination (RFE) recursively removing insignificant features to increase classification performance. In spite of SVM’s outstanding fortes in many applications, the current SVMs are only focused towards single data analysis, and so inevitably run into the problem of low reproducibility. To address this problem, we propose a meta-analytic framework based on the support vector machine (Meta-SVM). The proposed Meta-SVM is motivated by the recent meta-analytic method exploiting the meta-analytic logistic regression (Meta-logistic; [22]). To our best knowledge, no method has been introduced, which extends the SVMs to combining multiple studies in a meta-analytic fashion. Related to this, we develop a novel implementation strategy in spirit of Newton’s method to estimate parameters of the Meta-SVM. It is commonplace that the objective function of SVM is formed with the hinge loss and a range of penalty terms (e.g., *L*_1_-lasso, group lasso and etcs). Importantly we, however, adopts the sparse group lasso technique (i.e., both *L*_1_-lasso and group lasso, simultaneously) to capture both common and study specific genetic effects across all studies. The proposed method, on this ground, achieves the identical purpose of rOP [41] and AW [21], meta-logistic [22] whose feature selection allows to detect specific effects. In genomic applications, it cannot be emphasized enough that data integration analysis has proved its practical utility and has become commonplace to identify key regulators of cancer. Thus, many have paid attention to credible validation strategies that build on multiple studies [7, 35]. Besides, meta-analysis essentially aids to adjust tissue specific effects possibly distorting the analysis of individual datasets [21]. The optimization strategy to estimate, therefore, focuses on how to maneuver these two terms (*L*_1_-lasso and group lasso) in the formula. To overcome some of known traditional optimization rules (e.g., linear and quadratic programming), which mostly entails heavy computing tasks, we propose an approximation method to relax computational complexity in favor of concise implementation. The idea is to approximate the hinge loss including but not limited to penalty terms by a quadratic form, and thereby we can apply the classical coordinate descent algorithm to optimize the whole objective function.

The paper is outlined as follows. In Methods section, we introduce the meta-analytic method that builds on the support vector machine (Meta-SVM) and its implementation strategy at length. Simulation studies section shows experimental studies to benchmark performance of feature detection under various experimental scenarios. In Applications to real genomic data section, we demonstrate the advantages of Meta-SVM in two real data applications using publicly available omics data, and concluding remarks are presented in Concluding remark section. An R package “*metaSVM*” is publicly available online at author’s github page (https://sites.google.com/site/sunghwanshome/).

## Methods

### Meta-analytic support vector machine (Meta-SVM)

Consider *M* independent studies, consisting of *n* ^(*m*)^ subjects of *m*-th study for 1≤*m*≤*M*. Let $y_{i}^{(m)}$ be a scalar of binary phenotypes and $x_{i}^{(m)}=\left (x_{i1}^{(m)},\dots,x_{ip}^{(m)}\right)$ be a vector, each containing *p* common variables of the *i*-th subject for 1≤*i*≤*n* ^(*m*)^ and 1≤*m*≤*M*. We consider an objective function of the *L*_1_ support vector machine using the single *m*-th data set

1 $$ Q^{\lambda}\left(\beta^{(m)}\right) = \sum_{i=1}^{n^{(m)}} \left[ 1 - y_{i}^{(m)} f\left(x_{i}^{(m)};\beta^{(m)}\right) \right]_{+} + \lambda \sum_{j=1}^{p} | \beta_{j}^{(m)} |,  $$

where *λ*>0, $f\left (x_{i}^{(m)};\beta ^{(m)}\right) = \beta _{0}^{(m)} + \sum _{j=1}^{p} x_{ij}^{(m)} \beta _{j}^{(m)} $ for 1≤*i*≤*n* ^(*m*)^ and $\beta ^{(m)} = \left (\beta _{0}^{(m)}, \dots, \beta _{p}^{(m)} \right) \in \mathbb {R}^{p+1}$. Due to the linearity of $f\left (x_{i}^{(m)};\beta ^{(m)}\right)$, this is typically known as the linear support vector machine. And our major interest is to estimate the solution of *β* ^(*m*)^ that minimizes (1). By extension, in pursuit of integrating the *M* studies to a unified model, we propose the meta-analytic support vector machine that builds on multiple data via both group lasso and *L*_1_ lasso (a.k.a sparse group lasso):

2 $$ {}Q^{\lambda_{1},\lambda_{2}}(\beta)=\sum_{m=1}^{M} \sum_{i=1}^{n^{(m)}} \Big [ 1 - y_{i}^{(m)} f\left(x_{i}^{(m)};\beta^{(m)}\right) \Big ]_{+} + \lambda_{1} \sum_{j=1}^{p} \sqrt{\sum_{m=1}^{M}{\left(\beta_{j}^{(m)} \right)^{2}} }+ \lambda_{2} \sum_{m=1}^{M} \sum_{j=1}^{p} | \beta^{(m)}_{j} |,  $$

where *λ*_1_,*λ*_2_>0, *β*=(*β* ^(1)^,…,*β* ^(*M*)^). Here it is interesting to note that the group lasso penalty, $\sqrt {\sum _{m=1}^{M}{ \big (\beta _{j}^{(m)}}\big)^{2}}$ comes into play to integrate the effect size of the *j*-th variable across *M* data sets. Of note, the *L*_1_ lasso penalty encourages the sparsity within a group that potentially circumvents the all-in and all-out fashion. Thus, this property is in line with meta-analytic feature selection even when heterogeneous studies are present in analysis, since the sparse group lasso allows to accommodate both common effects across all studies and study specific effects simultaneously. Let

$$ \hat\beta^{(m)} = {\underset{{\beta^{(m)} \in \mathbb{R}^{p+1}}}{\text{argmin}}} Q^{\lambda_{1},\lambda_{2}} \left(\beta^{(m)}\right) $$

be the sparse group lasso estimator of the meta-analytic support vector machine for *m*-th study for 1≤*m*≤*M*.

### Implementation strategy

For estimating *β*, the SVM traditionally exploits the linear or quadratic programming well-suited to SVM’s dual problem. To our best knowledge, no coordinate descent-type optimization has yet been proposed to address the sparse group lasso problem despite the coordinate-type approach’s utility for implementation. The coordinate descent algorithm is one of the most popular algorithms that are built on the convexity assumption. To apply this algorithm to (2), an approximation to the smooth objective function is required on account of the non-differential property of the hinge loss and the group lasso penalty. With a little of algebraic trick, the group lasso penalty can be made twice-differentiable. Precisely, we add some sufficiently small constant inside the square root, in the way that the first and second derivative of the *L*_1_-lasso and group lasso penalty terms can be made at $\beta _{j}^{(m)}=0$. When it comes to the non-differential hinge loss, Zhang et al. [48] proposed the successive quadratic algorithm (SQA): a generalization of Newton’s method for unconstrained optimization such that it finds a step away from the current point of iteration by minimizing a quadratic approximation of the problem. Taken together, the objective function (2) can be approximated to

3 $$\begin{array}{*{20}l} {}\tilde{Q}^{\lambda_{1},\lambda_{2}}(\beta) = & \sum_{m=1}^{M} \left[ \frac 1 2 - \frac{1}{2n^{(m)}} \sum_{i=1}^{n^{(m)}} {y_{i}^{(m)} f(x_{i}^{(m)};\beta^{(m)})} + \frac{1}{4n^{(m)}} \sum_{i=1}^{n^{(m)}} {|y_{i}^{(m)} - f(x_{i}^{(m)};\beta^{(m)*})|}  \right.\\ &\left.+ \frac{1}{4n^{(m)}} \sum_{i=1}^{n^{(m)}} \frac{[y_{i}^{(m)} - f(x_{i}^{(m)};\beta^{(m)})]^{2}} {|y_{i}^{(m)} - f(x_{i}^{(m)};\beta^{(m)*})|}\right] + \lambda_{1} \sum_{j=1}^{p} \sqrt{\sum_{m=1}^{M}{\left(\beta_{j}^{(m)}\right)^{2}}}\\ &+ \lambda_{2} \sum_{m=1}^{M} \sum_{j=1}^{p} | \beta^{(m)}_{j} |, \end{array} $$

where *β* ^(*m*)∗^ is an estimated coefficient vector at the current point for 1≤*m*≤*M*. Contrary to (2), $\tilde {Q}^{\lambda _{1},\lambda _{2}}(\beta)$ is differentiable with respect to *β*, convex and separable with respect to all of variables so that we can apply the coordinate descent algorithm by means of Newton’s method. Update

4 $$ \beta_{j}^{(m)^{(t+1)}} \leftarrow \beta_{j}^{(m)^{(t)}} - \frac{\nabla \tilde{Q}^{\lambda_{1},\lambda_{2}} \left(\beta_{0}^{(m)^{(t+1)}}, \ldots, \beta_{j-1}^{(m)^{(t+1)}}, \beta_{j}^{(m)^{(t)}}, \ldots, \beta_{p}^{(m)^{(t)}}\right)_{j+1}} {\nabla^{2} \tilde{Q}^{\lambda_{1},\lambda_{2}} \left(\beta_{0}^{(m)^{(t+1)}}, \ldots, \beta_{j-1}^{(m)^{(t+1)}}, \beta_{j}^{(m)^{(t)}}, \ldots, \beta_{p}^{(m)^{(t)}}\right)_{j+1,j+1}}  $$

and iterate for 1≤*j*≤*p* and 1≤*m*≤*M* until convergence. More details are provided in Appendix.

## Simulation studies

To evaluate the performance of the proposed Meta-SVM method in the genomic setting, we simulated expression profiles with arbitrary correlated gene structures and variable effect sizes as follows: Simulate gene correlation structure for *P*=30 genes, *N*=20 samples in each study, and *M*=3. In each study, 10 out of 30 genes belong to *C*=2 independent clusters.

- Randomly sample gene cluster labels of 30 genes (*C*_*p*_∈{0,1,2} and 1≤*p*≤*P*), such that *C*=2 clusters each containing 5 genes are generated $(\Sigma _{p=1}^{P} 1 (C_{p} = c) = 5$, 1≤*c*≤*C*=2) and the remaining 20 genes are unclustered genes ($\Sigma _{p=1}^{P} 1(C_{p} = 0) = 20$).
- For any cluster *c* (1≤*c*≤*C*) in study *m* (1≤*m*≤*M*), sample ${\Sigma _{c}^{(m)}}^{*} \sim W^{-1}(\psi,60)$, where *ψ*=0.5*I*_5×5_+0.5*J*_5×5_, *W* ^−1^ denotes the inverse Wishart distribution, *I* is the identity matrix and *J* is the matrix with all the entries being 1. Set vector $\sigma _{c}^{(m)}$ as the square roots of the diagonal elements in ${\Sigma _{c}^{(m)}}^{*}$. Calculate $\sigma _{c}^{(m)}$ such that $\sigma _{c}^{(m)}\Sigma _{c}^{(m)} {\sigma _{c}^{(m)}}^{\top } = {\Sigma _{c}^{(m)}}^{*}$.
- Denote by $p_{1}^{(c)}, \cdots, p_{5}^{(c)}$ as the indices for genes in cluster *c*. In other words, $C_{p_{j}^{(c)}} = c$, where 1≤*c*≤2 and 1≤*j*≤5. Sample expression of clustered genes by $\big ({X^{(m)}_{p_{1}^{(c)}n}},\cdots,{X^{(m)}_{p_{5}^{(c)}n}})^{\top } \sim MVN(0, R \Sigma _{c}^{(m)})$, where 1≤*n*≤*N*=20, 1≤*m*≤*M* and *R* is an arbitrary constant for adjusting of total variance (*R*=1 as default). Sample expression for unclustered genes $X^{(m)}_{pn} \sim N(0, R)$ for 1≤*n*≤*N* and 1 ≤*m*≤*M* if *C*_*p*_=0.
- To simulate differential expression pattern, sample effect sizes $\mu _{p}^{(m)}$ from *Unif*(0.1,0.5) for 1≤*p*≤10 as differential expression (DE) genes and set $\mu _{p}^{(m)}=0$ for 11≤*p*≤*P* as non-DE genes.
- For the first 10 control samples, $Y_{pn}^{(m)} ={X^{(m)}_{pn}}(1 \leq p \leq P,1 \leq n \leq N/2=10,$ 1≤*m*≤*M*). For cases, $Y_{p(n+10)}^{(m)} ={X^{(m)}_{p(n+10)}} +\mu _{p}^{(m)} (1 \leq p \leq P, 1 \leq n \leq N/2=10,1 \leq m \leq M)$.

All tuning parameters (*λ*_1_ and *λ*_2_) are chosen by cross-validation, and the simulations were repeated 50 times. Table 1 summarizes the results of all simulation studies. It is noteworthy that the Meta-SVM achieves higher Youden index (= sensitivity + specificity −1) compare to the meta-logistic regression model across all experimental scenarios (i.e., *R*=0.1,0.3 and 0.5), and thus this suggests the Meta-SVM performs better in identifying the true signal features. Given that the meta-logistics model results in low sensitivity, the meta-logistic model has a tendency to overly penalize the effect size of features. In contrast, when data are sampled with low variance (*R*=0.1), specificity of the meta-logistic model is shown to be a little higher than that of the Meta-SVM (e.g., 1, 0.997 and 0.994 for the Meta-logistic, and 0.9843, 0.9837 and 0.9737 for the Meta-SVM), and yet the meta-logistic model still suffers low sensitivity at the expense of high specificity. Inspired by the simulation design introduced by meta-analysis of rth ordered p-value (rOP) [41], we also designed simulation schemes such that only a few studies provide major signals that differentiate binary outcomes like real data. To this end, we replaced signal genes of one or two studies with complete random noise (i.e., sampled from *N*(0,*R*); no signal genes). This leads to only one or two signal genes, respectively, among three data sets. Under this simulation scenario, the Meta-SVM still performs better as in Table 1, presenting higher Youden index than the meta-logistic model no matter how many random noises are imposed.

**Table 1:** Shown are the results of experimental studies to compare the meta-logistic model with the meta-analytic SVM

	Meta-SVM			Meta-logistic regression		
Variance (*R*)	Sensitivity (SE)	Specificity (SE)	Youden	Sensitivity (SE)	Specificity (SE)	Youden
No inclusion of random study						
0.1	0.828 (0.001)	0.9843 (0)	1.812	0.1073 (0)	1 (0)	1.107
0.3	0.8127 (0.002)	0.8707 (0.001)	1.683	0.2087 (0.001)	0.996 (0)	1.205
0.5	0.76 (0.002)	0.867 (0.001)	1.627	0.2633 (0.001)	0.9123 (0.001)	1.176
Inclusion of one random study						
0.1	0.8007 (0.011)	0.9837 (0.002)	1.784	0.102 (0.004)	0.997 (0.001)	1.099
0.3	0.6673 (0.013)	0.8497 (0.009)	1.517	0.2113 (0.009)	0.966 (0.005)	1.177
0.5	0.6013 (0.017)	0.852 (0.009)	1.453	0.2527 (0.01)	0.8667 (0.008)	1.119
Inclusion of two random studies						
0.1	0.624 (0.016)	0.9737 (0.009)	1.598	0.0847 (0.005)	0.994 (0.001)	1.079
0.3	0.51 (0.016)	0.8433 (0.006)	1.353	0.1727 (0.011)	0.9317 (0.005)	1.104
0.5	0.4167 (0.012)	0.85 (0.009)	1.267	0.256 (0.012)	0.8193 (0.006)	1.075

## Applications to real genomic data

In this section, we apply the Meta-SVM methods to two real examples of idiopathic pulmonary fibrosis expression profiles (IPF; 221 samples in four studies of binary outcome (i.e., case and control)) and breast cancer expression profiles provided by The Cancer Genome Atlas (TCGA) including mRNA, copy number variation (CNV) and epigenetic DNA methylation (http://cancergenome.nih.gov/; 300 samples of estrogen receptor binary outcome (i.e., ER+ and ER-)). It should be noticed that we integrate in the first application (IPF) four homogeneous studies in a fashion of horizontal integration, whereas we align in the second application (breast cancer) three genomic data by the common cohort in the context of vertical integration. Integrating multilevel-omics data is reasonable, in that inter-regulation flows in systems biology are present from CNV to mRNA and from DNA methylation to mRNA [16]. Therefore, these inter-omics features aligned on identical protein coding regions can be jointly estimated in the group lasso. Table 2 outlines the data descriptions, for a total of seven data sets and source references. In the pre-processing stage, genes and DNA methylation probes were matched across homogeneous studies and multi-omics data, and centered with scaling. Non-expressed and/or non-informative genes were filtered according to the rank sum of mean intensities and variances across studies. Importantly noted is that this filtering procedure has been used in a previous meta-analysis work [47] and this filtering step is unbiased since class labels are not involved in the process. This generated 110 common genes in IPF study and 108 common genes and matched methylation probes in TCGA for down-stream prediction analysis.

**Table 2:** Shown are the brief descriptions of the eight microarray datasets of disease-related binary phenotypes (e.g., case and control). All datasets are publicly available

Name	Study	Type	# of samples	Control	Case	Reference
TCGA	breast cancer	mRNA	300	234 (ER+)	66 (ER-)	The Cancer Genome Atlas (TCGA)
TCGA	breast cancer	Methylation	300	234 (ER+)	66 (ER-)	The Cancer Genome Atlas (TCGA)
TCGA	breast cancer	CNV	300	234 (ER+)	66 (ER-)	The Cancer Genome Atlas (TCGA)
KangA (batch 1)	IPF	mRNA	63	11	52	Kang *et al* (2012). GSE47460
KangB (batch 2)	IPF	mRNA	96	21	75	Kang et al. (2012) GSE47460
Konishi	IPF	mRNA	38	15	23	Konishi et al. (2009), GSE10667
Pardo	IPF	mRNA	24	11	13	Pardo et al. (2005), GSE2052

We applied gene set enrichment analysis to TCGA breast cancer data to figure out if our identified gene sets are in line with underlying biological pathways from the KEGG database [12]. It is notable that the identified gene set of the TCGA multiple omics data in Table 3 is significantly enriched in the ABC transporters pathways, which is already well-known to be correlated to breast cancer mechanisms, particularly related to estrogen receptor and drug resistance [8,28]. To our surprise, the ABC transporters pathway is considerably relevant to breast cancer mechanisms in many ways. For instance, breast cancer resistance protein (BCRP) is an ATP-binding cassette (ABC) transporter known as a molecular cause of multidrug resistance (MDR) in diverse cancer cells [46]. Besides Nakanishi et al. [28] discovered that up-regulation of BCRP mRNA expression was shown in estrogen receptor (ER)-positive breast cancer. This identified pathway has been consistently verified as critical for cancer outcomes and sensitivity to therapeutic treatments [8,19]. In previous study under the similar design [10], ABCC8 and ABCC11 in Table 3 are believed to be modifiers of progression and response to the chemotherapy of breast cancer.

**Table 3:** This table includes selected features of multiple omics data via the Meta-SVM

Four studies of lung disease (IPF)
C20orf114 MMP7 CXCL14 AGER TMEM100 THY1 CXCL2 HSD17B6 CCL18 CPA3 GEM
LEPREL1 ANXA3 CYP1B1 LRRC32 EMP2 FHL2 ADM C7 ITGA7 IGFBP2 BACE2 FKBP11
RGS5 FCGR3A SRPX FBLN2 HPCAL1 SOX4 CD248 CLDN5 LTBP1 ALOX5AP
Three multi-omics data of breast cancer (TCGA)
ABCC11 ABCC8 ACOX2 CAMP CST9L GRPR LAMP3 LCN2 LTF
MUCL1 NME5 THRSP VTCN1
- This gene set is significantly enriched in the ABC transporters (KEGG)
(FDR adjusted *p*-value = 0.025).

Generally idiopathic pulmonary fibrosis (IPF) is one of fatal lung diseases with a poor prognosis. Thus, it is quite imperative to monitor potential predictors of outcome. The original studies in Table 2 [17,32] posed a hypothesis on molecular biomarkers associated with IPF, and presented differentially expressed (DE) genes that distinguish IPF and control patients. For instance, Konishi et al. [17] identified in qRT-PCR microarray experiments MMP7, AGER and MMP7 are significantly higher and AGER is significantly lower in IPF. Pardo et al. [32] also pointed out that MMP7 is more significantly overexpressed compared with control lungs. Note that Meta-SVM is shown to be consistent with known evidence as detecting AGER and MMP7. Our findings in Table 3 also include CCL18. Importantly, it has been repeatedly reported that expression of CCL18 relates to course of pulmonary function parameters in patients with pulmonary fibrosis [33,34]. However, there was a little discrepancy regarding the roles of CCL18 according to the previous studies [31,33]. And yet, since the Meta-SVM incorporates multiple data together, we can still give more credence to CCL18 as a molecular biomarker to predict IPF.

Of the 33 identified genes of IPF data (See Table 3 and Additional file 1: Table S1), we further reduce the number of genes for post-hoc analysis by exploring significant gene modules, equivalently gene-gene interaction, via Netbox [6]. NetBox is an analytic software well-suited to detect connecting genes to a network, identifying statistically significant “linker” genes on the basis of four public data sources: NCI-Nature Pathway Interaction Database [40], Human Protein Reference Database [13], MSKCC Cancer Cell Map (http://www.mskcc.org/), and Reactome [11]. We implemented gene-gene interaction analysis, and successfully detected four gene modules, each of which constitutes mutually correlated genes. Additional file 1: Figure S1 displays the structure of combined networks based on four distinct gene modules. Focusing on the genes that belong to the four modules, we examine on MMP7 [32,44,50], LTBP1 [20], FHL2 [1], CXCL2 [42], THY1 [39] and AGER [17] to confirm whether or not these are associated with IPF (See Additional file 1: Table S3). MMP7 is traditionally thought of as the predictive signature since MMP7 of IPF patients is among the molecules that are more significantly overexpressed compared with control lungs [32]. More interestingly, Bauer et al. [1] identified a novel set of 12 disease-relevant translational gene markers including FHL2, MMP7 that are able to separate almost all patients with IPF from control subjects in multiple large-scale cohorts. Related to CXCL2, [42] investigated the pathogenesis of pulmonary fibrosis relevant to the imbalance in the expression of these angiogenic and angiostatic CXC chemokines. This study demonstrates in the bleomycin model that the amount of CXCL2 is found positively correlated with measures of fibrosis. When it comes to novel therapeutic targets, profiling DNA methylation changes to fibrosis has been increasingly spotlighted by observing hypomethylation of oncogene promoters. In doing so, Sanders et al. [39] reported that hypermethylation epigenetically decreases THY1 (See Additional file 1: Table S3) in IPF fibroblasts as IPF suppressor genes. Taken together, the Meta-SVM is found to be efficient in identifying potential biomarkers that facilitate elucidating the disease process.

## Concluding remark

In this article, we introduce a meta-analytic framework using the support vector machine. The objective function of Meta-SVM applies the hinge loss and the sparse group lasso, and so we also develop a novel strategy for implementing the sparse group lasso in the context of Newton’s method. More importantly, the proposed Meta-SVM shows many advantages in discovering the underlying true signals and in detecting gene sets enriched for cancer disease process validated as biologically significant. Putting all things together, we conclude that the proposed meta-SVM is a reasonable choice to effectively achieve the common aims of meta-analysis. This is not that surprising given that the Meta-SVM takes advantages of the meta-analytic design that jointly leverages multiple omics data. For future study, we may improve computational speed via low-level programming languages (e.g., C/C++ or Fortran) since coordinate descent algorithm sometimes leads to heavy computation due to slow convergence at the exchange of the straightforward algorithm structure. Usage of diverse kernels (e.g., quadratic and radial basis kernels) can be a possible choice to improve performance of feature discovery, and prediction accuracy. Moreover, it is worthwhile to impose interaction terms in the model, making it possible to account for the complex association among genomic features. We leave these ideas for future tasks.

## Appendix

### Optimization of a penalized univariate quadratic function

#### Univariate Lasso problem

Consider a quadratic function *q* defined as

$$ q(z) = \frac b2 (z - c)^{2} + d \quad\text{for}~z \in \mathbb{R}, $$

where *b*>0 and $c, d \in \mathbb {R}$. Let *q* ^*λ*^ be a penalized quadratic function given as

$$ q^{\lambda}(z) = q(z) + \lambda {\left| z \right|} \quad\text{for}~z \in \mathbb{R} $$

and denote

$$ z^{\lambda} = \underset{z \in \mathbb{R}}{{\arg\!\min}} \, q^{\lambda}(z). $$

Note that $ b = q''(z) \quad \forall z \in \mathbb {R} $ and $c = \text {argmin}_{z \in \mathbb {R}}q(z)$ since *c* is the solution to *q* ^′^(*z*)=0.

##### **Theorem 1**

The minimizer *z* ^*λ*^ of *q* ^*λ*^ is given by

5 $$ z^{\lambda} = \mathsf{ST} \left(c, \frac{\lambda}{b} \right),  $$

where the soft-thresholding operator is defined by

$$\mathsf{ST}(y, \lambda) = \begin{cases} y - \lambda & \text{if} \quad y > \lambda \\ y + \lambda & \text{if} \quad y < -\lambda \\ 0 & \text{if} \quad {\left| y \right|} \le \lambda \\ \end{cases} $$

for $y \in \mathbb {R}$ and *λ*>0.

#### Univariate Sparse group lasso problem

Let

6 $$ q^{\lambda_{1}, \lambda_{2}}(z) = \frac{b}{2} (z - c)^{2} + \lambda_{1} \sqrt{z^{2} + d} + \lambda_{2} {\left| z \right|} \quad\text{for} \,z \in \mathbb{R},  $$

where *b*>0, *d*≥0 and $c \in \mathbb {R}$. If *d*=0, then the univariate sparse group lasso problem becomes the univariate lasso problem. Equivalently,

$$q^{\lambda_{1}, \lambda_{2}}(z) = \frac{b}{2} (z - c)^{2} + (\lambda_{1} + \lambda_{2}) {\left| z \right|} \quad\text{for} \,z \in \mathbb{R} $$

and we have

$$z^{\lambda_{1}, \lambda_{2}} = \mathsf{ST} \left(c, (\lambda_{1} + \lambda_{2}) / b \right). $$

Consider the univariate sparse group lasso problem with *d*>0. Let *F*_*s*_(*z*) be the form of the cdf of the logistic distribution with a scale parameter *s*>0, which is given by

$$ F_{s}(z) = 2 \left(\frac{\exp(z/s)}{1 + \exp(z/s)} \right) - 1. $$

An approximation to $q^{\lambda _{1}, \lambda _2}\phantom {\dot {i}\!}$ is

$$\tilde q^{\lambda_{1}, \lambda_{2}}(z) = \frac{b}{2} (z - c)^{2} + \lambda_{1} \sqrt{z^{2} + d} + \lambda_{2} \int_{-\infty}^{z} F_{s}(u) du \quad\text{for}~z \in \mathbb{R}. $$

When *s* is sufficiently small, $\tilde z^{\lambda _{1}, \lambda _{2}}= \text {argmin}_{z \in \mathbb {R}} \tilde q^{\lambda _{1}, \lambda _2}(z)$ is close to

$$ z^{\lambda_{1}, \lambda_{2}} = \underset{z \in \mathbb{R}}{\text{argmin}}\, q^{\lambda_{1}, \lambda_{2}}(z). $$

Using the Newton-Raphson method, we can find $ \tilde z^{\lambda _{1}, \lambda _2} $. Note

$$\frac{d\tilde q^{\lambda_{1}, \lambda_{2}}(z)}{dz} = b (z - c) + \lambda_{1} \frac {z}{\sqrt{z^{2} + d}} + \lambda_{2} F_{s}(z) \quad\text{for} \,z \in \mathbb{R} $$

and

$$\frac{d^{2}\tilde q^{\lambda_{1}, \lambda_{2}}(z)}{dz^{2}} = b + \lambda_{1} \frac{d}{(\sqrt{z^{2} + d})^{3}} + \lambda_{2} w_{s}(z) \quad\text{for}\, z \in \mathbb{R} $$

where

$$w_{s}(z) = \frac{1}{2s} F_{s}(z) \left(1 + F_{s}(z) \right). $$

Starting from an initial value *z* ^(0)^, we iterate

$$ z^{(t+1)} = z^{(t)} - \dfrac {d\tilde q^{\lambda_{1}, \lambda_{2}}(z^{(t)})/dz} {d^{2}\tilde q^{\lambda_{1}, \lambda_{2}}(z^{(t)})/dz^{2}}. $$

### Implementation for the meta-analytic SVM

In order to estimate the solution of *β* ^(*m*)^, we approximate (3) to the univariate quadratic function, and then apply the Newton-Raphson method. To derive the quadratic form, we revisit the successive quadratics algorithm [48]. For each 1≤*i*≤*n* ^(*m*)^ and 1≤*m*≤*M*, we have $\big (y^{(m)}_{i} \big)^2=1$ and

7 $$ \left[ 1 - y^{(m)}_{i} f\left(x_{i}^{(m)};\beta^{(m)}\right) \right]_{+} = \frac{1 - y^{(m)}_{i} f\left(x_{i}^{(m)};\beta^{(m)}\right)}{2} + \frac {\left|y^{(m)}_{i} - f\left(x_{i}^{(m)};\beta^{(m)}\right)\right|}{2}  $$

Assume $\beta ^{(m)^{*}}$ is given, we consider the local quadratic approximation for the second term in (7):

$$  \left| y^{(m)}_{i} - f\left(x_{i}^{(m)};\beta^{(m)}\right) \right| \approx \frac{1}{2} \frac{\left[ y^{(m)}_{i} - f\left(x_{i}^{(m)};\beta^{(m)}\right) \right]^{2}} {\left| y^{(m)}_{i} - f\left(x_{i}^{(m)};\beta^{(m)^{*}}\right) \right|} + \frac{1}{2} \left| y^{(m)}_{i} - f\left(x_{i}^{(m)};\beta^{(m)^{*}}\right) \right|,   $$

where *β* ^(*m*)∗^ is an estimated coefficient vector at the current point. The quadratic form approximated to the entire objective function (3).

Given $\tilde \beta ^{(m)} = \left (\tilde \beta _{0}^{(m)}, \ldots, \tilde \beta _{p}^{(m)}\right) \in \mathbb {R}^{p+1}$, the function $\tilde {Q}^{\lambda _{1},\lambda _2}\left (\tilde \beta _{0}^{(m)}, \ldots, \tilde \beta _{j-1}^{(m)},\right.$ $\left.\beta _{j}^{(m)}, \tilde \beta _{j+1}^{(m)}, \ldots, \tilde \beta _{p}^{(m)}\right)$ is an univariate sparse group quadratic function of the form (6) with argument $z = \beta _{j}^{(m)}$ with suitable *b*,*c*,*d*. We update $\beta _{j}^{(m)}$ by the minimizer of $\tilde {Q}^{\lambda _{1},\lambda _2}\left (\tilde \beta _{0}^{(m)}, \ldots, \tilde \beta _{j-1}^{(m)}, \beta _{j}^{(m)}, \tilde \beta _{j+1}^{(m)}, \ldots, \tilde \beta _{p}^{(m)}\right)$ for 0≤*j*≤*p* and 1≤*m*≤*M*. Let

$$\mathsf{X}^{(m)} =\left[ \begin{array}{cccc} 1 & x_{11}^{(m)} & \ldots & x_{1p}^{(m)} \\ \vdots & \vdots & \ddots & \vdots \\ 1 & x_{{n^{(m)}}1}^{(m)} & \ldots & x_{{n^{(m)}}p}^{(m)} \end{array} \right] \in \mathbb{R}^{n^{(m)} \times (p+1)}, \quad \mathsf{y}^{(m)} = \left[ \begin{array}{c} y_{1}^{(m)} \\ \vdots \\ y_{n^{(m)}}^{(m)} \end{array} \right] \in \mathbb{R}^{n^{(m)}}, $$

$$\mathsf{Z}^{(m)} = \left[ \begin{array}{ccccc} y_{1}^{(m)}& y_{1}^{(m)} x_{11}^{(m)} & \ldots & y_{1}^{(m)} x_{1p}^{(m)} \\ \vdots & \vdots & \ddots & \vdots \\ y_{n}^{(m)} & y_{n^{(m)}}^{(m)} x_{{n^{(m)}}1}^{(m)} & \ldots & y_{n^{(m)}}^{(m)} x_{{n^{(m)}}p}^{(m)} \end{array} \right] \in \mathbb{R}^{n^{(m)} \times (p+1)} $$

and

$$\mathsf{W}^{(m)} = \text{diag}{\left(w_{1}^{(m)}, \ldots, w_{n^{(m)}}^{(m)} \right)} = \left[ \begin{array}{lll} w_{1}^{(m)} & & \\ & \ddots & \\ & & {w_{n^{(m)}}^{(m)}} \end{array} \right] \in \mathbb{R}^{n^{(m)} \times n^{(m)}}, $$

where

$$ w_{i}^{(m)} = {\left| y_{i}^{(m)} - f(x_{i}^{(m)};\beta^{(m)^{*}}) \right|}^{-1} \quad\text{for}\, i = 1, \ldots, n^{(m)}. $$

Observe

$$\sum_{i=1}^{n^{(m)}} {y_{i}^{(m)} f(x_{i}^{(m)};\beta^{(m)})} = \mathsf{1}^{\top} \mathsf{Z}^{(m)} \beta^{(m)} \quad\text{for} \, \mathsf{1} = \left[ \begin{array}{l} 1 \\ \vdots \\ 1 \end{array} \right]\in \mathbb{R}^{n^{(m)}} $$

and

$$\begin{aligned} &\sum_{i=1}^{n} \frac{\left[y^{(m)}_{i} - f(x_{i}^{(m)};\beta^{(m)}) \right]{~}^{2}}{{\left| y^{(m)}_{i} - f(x_{i}^{(m)};\beta^{(m)^{*}}) \right|}}\\ &= \left(\mathsf{y}^{(m)} - \mathsf{X}^{(m)}\beta^{(m)}\right){~}^{\top} \mathsf{W}^{(m)} \left(\mathsf{y}^{(m)} - \mathsf{X}^{(m)}\beta^{(m)}\right) \\ &= {\mathsf{y}^{(m)}}^{\top} \mathsf{W}^{(m)} \mathsf{y}^{(m)} - 2 {\beta^{(m)}}^{\top} {\mathsf{X}^{(m)}}^{\top} \mathsf{W}^{(m)} \mathsf{y}^{(m)} + {\beta^{(m)}}^{\top} {\mathsf{X}^{(m)}}^{\top} \mathsf{W}^{(m)} \mathsf{X}^{(m)} \beta^{(m)}. \end{aligned} $$

Combining these, we obtain

8 $$\begin{array}{*{20}l} {}\tilde{Q}^{\lambda_{1},\lambda_{2}}\left(\beta^{(m)}\right) =  &- \frac{1}{2n^{(m)}} \mathsf{1}^{\top} \mathsf{Z}^{(m)} \beta^{(m)} \\ &+ \frac{1}{4n^{(m)}} \left({\mathsf{y}^{(m)}}^{\top} \mathsf{W}^{(m)} \mathsf{y}^{(m)} - 2 {\beta^{(m)}}^{\top} {\mathsf{X}^{(m)}}^{\top} \mathsf{W}^{(m)} \mathsf{y}^{(m)}\right. \\ &\left. + {\beta^{(m)}}^{\top} {\mathsf{X}^{(m)}}^{\top} \mathsf{W}^{(m)} \mathsf{X}^{(m)} \beta^{(m)} \right)  \\ & + \lambda_{1} \sum_{j=1}^{p} \sqrt{\sum_{m=1}^{M}\big (\beta_{j}^{(m)}\big)^{2}} + \lambda_{2} \sum_{m=1}^{M} \sum_{j=1}^{p} {\left| \beta^{(m)}_{j} \right|}. \end{array} $$

The gradient and the Hessian matrix of $\tilde {Q}^{\lambda _{1}, \lambda _2}$ are, respectively, given as

9 $${}  \nabla \tilde{Q}^{\lambda_{1},\lambda_{2}}\left(\beta^{(m)}\right) = - \frac{1}{2n^{(m)}} \left[ {\mathsf{X}^{(m)}}^{\top} \mathsf{W}^{(m)} \left(\mathsf{y}^{(m)} - \mathsf{X}^{(m)}\beta^{(m)}\right) + {\mathsf{Z}^{(m)}}^{\top} \mathsf{1} \right] + \lambda_{1} \mathsf{B}_{1}^{\prime} + \lambda_{2} \mathsf{B}_{2}^{\prime},  $$

and

10 $$ \nabla^{2} \tilde{Q}^{\lambda_{1},\lambda_{2}}\left(\beta^{(m)}\right) = \frac{1}{2n^{(m)}} {\mathsf{X}^{(m)}}^{\top} \mathsf{W}^{(m)} \mathsf{X}^{(m)} + \lambda_{1} \mathsf{B}_{1}^{\prime\prime} + \lambda_{2} \mathsf{B}_{2}^{\prime\prime},  $$

where

$${\kern50pt}\mathsf{B}_{1}^{\prime} = \left[ \begin{array}{c} 0 \\ \frac {\beta_{1}^{(m)}} {\sqrt{{\beta_{1}^{(m)^{2}}+d_{1}+\epsilon}}} \\ \vdots \\ \frac {\beta_{p}^{(m)}} {\sqrt{{\beta_{p}^{(m)^{2}}+d_{p}+\epsilon}}} \end{array} \quad \right], \mathsf{B}_{1}^{\prime\prime} = \left[ \begin{array}{c}0 \\ \frac {d_{1}} {\left(\sqrt{{\beta_{1}^{(m)^{2}}+d_{1}+\epsilon}}\right)^{3}} \\ \vdots \\ \frac {d_{p}} {\left(\sqrt{{\beta_{p}^{(m)^{2}}+d_{p}+\epsilon}}\right)^{3}} \\ \end{array} \right], $$

$${\kern50pt}\mathsf{B}_{2}^{\prime} = \left[ \begin{array}{c} 0 \\ F_{s}\left(\beta_{1}^{(m)}\right) \\ \vdots \\ F_{s}\left(\beta_{p}^{(m)}\right) \end{array} \right], \quad \mathsf{B}_{2}^{\prime\prime} = \left[ \begin{array}{c} 0 \\ w_{s}\left(\beta_{1}^{(m)}\right) \\ \vdots \\ w_{s}\left(\beta_{p}^{(m)}\right) \end{array} \right], $$

$d_{j} = \sum _{k \neq j} \beta _{k}^{(m)^{2}}$ and a sufficiently small positive constant *ε* for 1≤*j*≤*p*. We propose the following algorithm to solve the meta-analytic SVM via Newton’s method in a fashion of coordinate descent algorithm:

**Table 4:** An algorithm for the meta-analytic SVM via Newton’s method

Step 1: For 1≤*m*≤*M*, set the initial value $\beta ^{(m)^{(0)}}$.
Step 2: Set *t*=0 and minimize $\tilde {Q}^{\lambda _{1},\lambda _{2}}\left (\beta _{j}^{(m)}\right)$ via Newton’s method:
$\beta _{j}^{(m)^{(t+1)}} \leftarrow \beta _{j}^{(m)^{(t)}} - \frac {\nabla \tilde {Q}^{\lambda _{1},\lambda _{2}} \left (\beta _{0}^{(m)^{(t+1)}}, \ldots, \beta _{j-1}^{(m)^{(t+1)}}, \beta _{j}^{(m)^{(t)}}, \ldots, \beta _{p}^{(m)^{(t)}}\right)_{j+1}} {\nabla ^{2} \tilde {Q}^{\lambda _{1},\lambda _{2}} \left (\beta _{0}^{(m)^{(t+1)}}, \ldots, \beta _{j-1}^{(m)^{(t+1)}}, \beta _{j}^{(m)^{(t)}}, \ldots, \beta _{p}^{(m)^{(t)}}\right)_{j+1,j+1}}$
for 0≤*j*≤*p* and 1≤*m*≤*M*.
Step 3: Update *t*=*t*+1 and go to Step 2 until convergence.

## Additional file

Additional file 1
**Table S1.** The Meta-SVM’s coefficient of lung disease mRNA data. **Table S2.** The Meta-SVM’s coefficient of TCGA breast cancer multi-level omics data. **Table S3.** Gene-gene interaction analysis using 33 identified genes of IPF mRNA data. **Figure S1.** Gene networks that display the relationships among significant genes. The orange nodes are the selected linker genes out of 33 genes in Table 3. The blue nodes indicate linker genes not presented in the original input list, but are significantly connected to members of the input list. (DOCX 187 kb)
