# Erratum to: Meta-analytic support vector machine for integrating multiple omics data

**DOI:** 10.1186/s13040-017-0128-6

**Published:** 2017-02-14

**Authors:** SungHwan Kim, Jae-Hwan Jhong, JungJun Lee, Ja-Yong Koo

**Affiliations:** 1 Department of Statistics, Korea University, Anam-dong, 136-701 Seoul, South Korea; 2 Department of Statistics, Keimyung University, Dalseoku, 42601 Daegu, South Korea

## Erratum

After publication of the original article [1], the author noticed errors to Table 1. The errors occurred in the fourth and seventh columns labelled ‘Youden’. The correct version of Table 1 is included in this erratum.

**Table 1:** Shown are the results of experimental studies to compare the meta-logistic model with the meta-analytic SVM

	Meta-SVM			Meta-logistic regression		
Variance (R)	Sensitivity (SE)	Specificity (SE)	Youden	Sensitivity (SE)	Specificity (SE)	Youden
	No inclusion of random study					
0.1	0.828 (0.001)	0.9843 (0)	0.812	0.1073 (0)	1 (0)	0.107
0.3	0.8127 (0.002)	0.8707 (0.001)	0.683	0.2087 (0.001)	0.996 (0)	0.205
0.5	0.76 (0.002)	0.867 (0.001)	0.627	0.2633 (0.001)	0.9123 (0.001)	0.176
	Inclusion of one random study					
0.1	0.8007 (0.011)	0.9837 (0.002)	0.784	0.102 (0.004)	0.997 (0.001)	0.099
0.3	0.6673 (0.013)	0.8497 (0.009)	0.517	0.2113 (0.009)	0.966 (0.005)	0.177
0.5	0.6013 (0.017)	0.852 (0.009)	0.453	0.2527 (0.01)	0.8667 (0.008)	0.119
	Inclusion of two random studies					
0.1	0.624 (0.016)	0.9737 (0.009)	0.598	0.0847 (0.005)	0.994 (0.001)	0.079
0.3	0.51 (0.016)	0.8433 (0.006)	0.353	0.1727 (0.011)	0.9317 (0.005)	0.104
0.5	0.4167 (0.012)	0.85 (0.009)	0.267	0.256 (0.012)	0.8193 (0.006)	0.075
