# Supplementary material for: Meta-analytic support vector machine for integrating multiple omics data
Source: BioData Min. 2017 Jan 26;10:2. doi: 10.1186/s13040-017-0126-8 (PMC5270233; doi:10.1186/s13040-017-0126-8)

**Supplementary Materials**

**Table S1: The Meta-SVM’s coefficient of lung disease mRNA data.**

| Gene symbol | KangA | KangB | Konishi | Pardo |
| --- | --- | --- | --- | --- |
| (intercept) | 0.76914 | 0.80380 | 0.83032 | 0.40852 |
| C20orf114 | -0.00012 | 0.00300 | 0.04199 | 0.05026 |
| MMP7 | 0.05584 | 0.04180 | 0.11346 | 0.05706 |
| CXCL14 | 0.02167 | 0.01244 | 0.05805 | 0.03685 |
| AGER | -0.00090 | -0.00231 | -0.02664 | -0.15232 |
| TMEM100 | -0.00026 | -0.00034 | -0.00004 | -0.00873 |
| THY1 | 0.12934 | 0.11255 | 0.26863 | 0.55016 |
| CXCL2 | -0.00002 | 0.00005 | -0.00304 | -0.00369 |
| HSD17B6 | -0.02504 | -0.00468 | -0.04257 | -0.02359 |
| CCL18 | 0.00032 | 0.00054 | 0.00187 | 0.00059 |
| CPA3 | 0.00126 | 0.02874 | 0.00155 | 0.00002 |
| GEM | 0.01788 | 0.01588 | 0.00001 | 0.00523 |
| LEPREL1 | -0.00277 | -0.02140 | -0.04041 | -0.04925 |
| ANXA3 | -0.00306 | -0.00007 | -0.00565 | -0.04140 |
| CYP1B1 | 0.00005 | -0.00016 | 0.04719 | 0.02212 |
| LRRC32 | -0.03978 | -0.00759 | -0.00941 | -0.03436 |
| EMP2 | -0.01583 | -0.00003 | -0.03368 | -0.02896 |
| FHL2 | 0.04112 | 0.00902 | 0.04500 | 0.03658 |
| ADM | -0.03825 | -0.07352 | -0.00241 | -0.03083 |
| C7 | 0.00871 | 0.00002 | 0.02721 | 0.00752 |
| ITGA7 | 0.01239 | 0.00001 | 0.04666 | 0.01963 |
| IGFBP2 | 0.00835 | 0.00452 | 0.01740 | 0.00885 |
| BACE2 | 0.00603 | 0.00021 | 0.00846 | 0.00190 |
| FKBP11 | 0.00017 | 0.00001 | 0.00060 | 0.00017 |
| RGS5 | 0.08753 | 0.01229 | 0.00002 | 0.01809 |
| FCGR3A | -0.00872 | 0.00001 | 0.00075 | -0.00125 |
| SRPX | -0.00088 | -0.00494 | 0.01859 | -0.00361 |
| FBLN2 | 0.03474 | 0.02529 | 0.04249 | 0.01470 |
| HPCAL1 | -0.00999 | -0.00553 | -0.00175 | -0.02279 |
| SOX4 | 0.06182 | 0.06976 | 0.00399 | -0.00440 |
| CD248 | 0.00391 | 0.00034 | 0.00400 | 0.00077 |
| CLDN5 | -0.07438 | -0.02519 | -0.00978 | -0.02728 |
| LTBP1 | 0.05831 | 0.07827 | 0.05555 | 0.04046 |
| ALOX5AP | -0.00004 | -0.00003 | 0.00002 | -0.00009 |

**Table S2: The Meta-SVM’s coefficient of TCGA breast cancer multi-level omics data.**

| Gene symbol | mRNA | Methylation | CNV |
| --- | --- | --- | --- |
| (intercept) | 0.40852 | 0.23082 | 0.50218 |
| ABCC11 | 0.00910 | -0.00002 | -0.00480 |
| ABCC8 | 0.01311 | -0.00001 | 0.00435 |
| ACOX2 | 0.03436 | -0.10025 | 0.05108 |
| CAMP | -0.00005 | -0.00169 | 0.00383 |
| CST9L | 0.00018 | 0.24501 | -0.12984 |
| GRPR | 0.06396 | -0.00576 | 0.00078 |
| LAMP3 | -0.00039 | 0.03265 | -0.08505 |
| LCN2 | -0.16257 | 0.01766 | 0.00001 |
| LTF | -0.00008 | -0.00002 | 0.00757 |
| MUCL1 | 0.02122 | 0.00795 | 0.05125 |
| NME5 | 0.00970 | -0.00019 | 0.11841 |
| THRSP | -0.02873 | -0.03897 | -0.00066 |
| VTCN1 | -0.00001 | 0.73054 | -0.17096 |

**Table S3: Gene-gene interaction analysis using 33 identified genes of IPF mRNA data.**

| Modules | # of genes | Included genes | | | | | | |
| --- | --- | --- | --- | --- | --- | --- | --- | --- |
| 0 | 2 | MMP7 | BCAN* |  |  |  |  |  |
| 1 | 4 | FBLN2 | LTBP1 | FBN1* | ITGB5* |  |  |  |
| 2 | 4 | FHL2 | FHL3* | LAMA1* | ITGA7 |  |  |  |
| 3 | 7 | PLG* | C7 | CXCL2 | ITGAM* | THY1 | AGER | ITGB2* |
| 4 | 2 | HPCAL1 | SLC34A1* |  |  |  |  |  |

**Figure S1: Gene networks that display the relationships among significant genes. The orange nodes are the selected linker genes out of 33 genes in Table 3. The blue nodes indicate linker genes not presented in the original input list, but are significantly connected to members of the input list.**


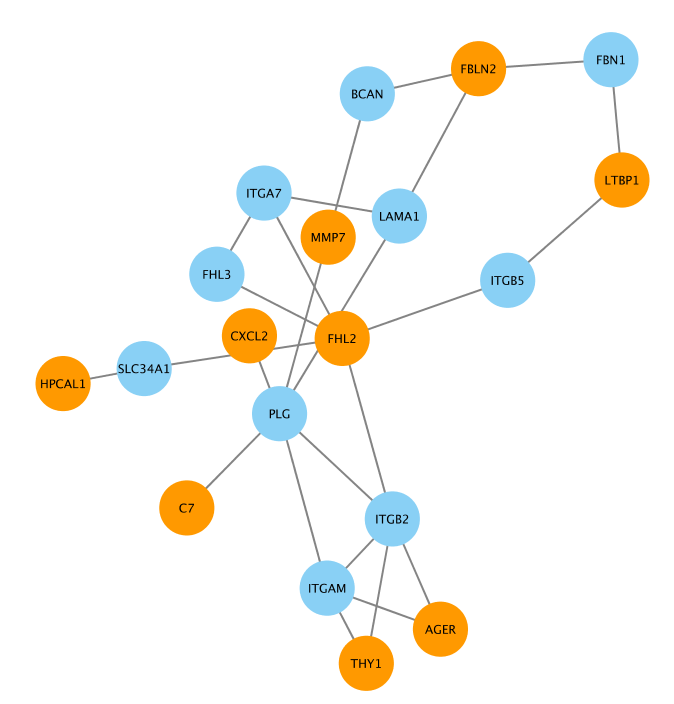

Supplement: Additional file 1 — Table S1. The Meta-SVM’s coefficient of lung disease mRNA data. Table S2. The Meta-SVM’s coefficient of TCGA breast cancer multi-level omics data. Table S3. Gene-gene interaction analysis using 33 identified genes of IPF mRNA data. Figure S1. Gene networks that display the relationships among significant genes. The orange nodes are the selected linker genes out of 33 genes in Table 3. The blue nodes indicate linker genes not presented in the original input list, but are significantly connected to members of the input list. (DOCX 187 kb) [file 13040_2017_126_MOESM1_ESM.docx]
